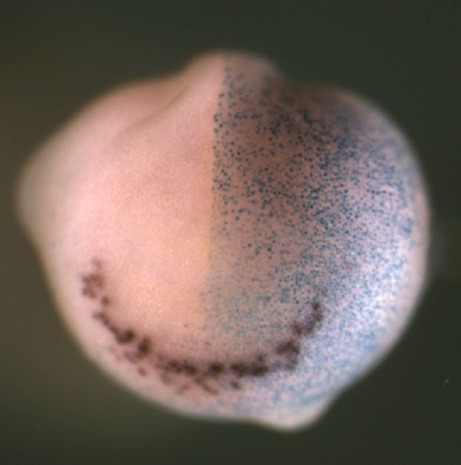# *Xenopus* as a developmental model of neuroblastoma

**Published:** 2015-05-01

Neuroblastoma (NB) is a paediatric form of cancer derived from the sympathetic nervous system. Recent genome-wide sequencing data suggest that often NB does not have a clear genetic cause, leading the authors to hypothesize that NB results from aberrations of normal development. To test this hypothesis, Anna Philpott's group used a population of anteroventral noradrenergic (AVNA) cells from *Xenopus* embryos. These cells share several features with mammalian sympathetic neurons, including the expression of noradrenergic-associated genetic markers such as the achaete-scute complex-like 1 (*Ascl1*) gene, which encodes a transcriptional driver of neurogenesis. By comparing AVNA and NB cells, the authors found that, whereas Ascl1 is only transiently expressed in AVNA cells, it is aberrantly maintained in NB, where it is phosphorylated on multiple serine-proline sites. The authors then show that differentiation of AVNA cells is enhanced by dephosphorylated Ascl1. Moreover, this process is inhibited by experimental manipulations of NB-associated genes, but, interestingly, dephosphorylation of Ascl1 is able to overcome this inhibition. This work demonstrates that *Xenopus* AVNA cells represent a unique system to study sympathetic nervous system development and its relationship to NB. Moreover, it suggests that Asc11 phosphorylation might promote stalled differentiation leading to NB, thus identifying a potential target for therapeutic purposes. **Page 429**

**Figure:**